# Liver-Specific Overexpression of Gamma-Glutamyltransferase Ameliorates Insulin Sensitivity of Male C57BL/6 Mice

**DOI:** 10.1155/2017/2654520

**Published:** 2017-06-04

**Authors:** Yang Long, Dan Jia, Libin Wei, Yumei Yang, Haoming Tian, Tao Chen

**Affiliations:** ^1^Department of Endocrinology and Metabolism, West China Hospital of Sichuan University, Chengdu 610041, China; ^2^Laboratory of Endocrinology, Experimental Medicine Center, The Affiliated Hospital of Southwest Medical University, Luzhou 646000, China; ^3^Division of General Practice, West China Hospital of Sichuan University, Chengdu 610041, China; ^4^Department of Stomatology, Hebei Medical University Affiliated North China Petroleum Bureau General Hospital, Renqiu 062552, China; ^5^Health Examination Management Center, Sichuan Province People's Hospital, Chengdu 610072, China

## Abstract

In the current study, we developed a liver-specific GGT-overexpressing mice model by rapid injection pLIVE-GGT vector through tail vein and investigated the effects of GGT elevation on glucose metabolism and insulin sensitivity. The serum GGT activity was significantly increased after 7 days of pLIVE-GGT1 vector delivery and lasted for about 3 weeks. GGT overexpression reduced the levels of GSSG and GSH in the liver and serum and had no effects on total antioxidative capacity in the liver, kidney, and skeletal muscle except for the pancreas. Increased GGT activity had no effect on the glucose tolerance but could facilitate blood glucose lowering after intraperitoneal insulin administration. The results of Western blotting showed that increased GGT activity enhanced insulin-induced AKT phosphorylation at Ser473. Furthermore, GGT inhibitor could attenuate the changes of insulin-induced blood glucose uptake and AKT phosphorylation in the liver. In summary, the present study developed a liver-specific GGT-overexpressing mice model and found that GGT elevation in short term had no effects on glucose metabolism but could increase insulin sensitivity through enhancing the activity of insulin signaling pathway.

## 1. Introduction

The prevalence of type 2 diabetes (T2D) is increasing worldwide, while the underlining mechanism is not fully elucidated. Recently, several prospective studies and meta-analyses suggested that gamma-glutamyltransferase (GGT), a previously recognized marker of alcoholic drinking and fatty liver, could predict the risk of T2D [1–3]. Such an association existed even when GGT was at physiologic level [4], in nonalcoholic drinkers and subjects without nonalcoholic fatty liver disease (NAFLD) [5, 6]. One study suggested that BMI could predict T2D only when GGT was at physiologic high levels [7].

GGT exists on the surface of nearly all kinds of epithelial cells and plays a critical role in regulating reactive oxygen species (ROS) level through balancing reduced glutathione (gamma-glutamyl-cysteinyl-glycine, GSH) and oxidized form glutathione disulfide (GSSG). The nature substrate of GGT is GSH, and the gamma-glutamyl of GSH can only be cleaved by GGT. GGT broke down GSH in extracellular fluids [5]. This process demands the cooperation of Fe(III) and will lead to the production of the superoxide anion and hydrogen peroxide [8]. Theoretically, increased GGT activity would result in altered levels of GSSG/GSH and overproduction of ROS, thus changing the oxidative status. Our previous study found that increased GGT activity combined with ferritin levels was linked to increased risk of T2D, and the mechanism might be related to increased oxidative stress [9]. Furthermore, other studies showed that elevated serum GGT concentration could be associated with islet beta-cell function and/or insulin resistance [10, 11].

However, the associations between elevated serum GGT and T2D, insulin resistance, and islet beta-cell function were built on epidemiological observational studies. In these studies, GGT elevation usually was accompanied by NALFD, ferritin, and other markers of oxidative stress and chronic inflammation [9, 12, 13]. Therefore, it is difficult to deduce whether causative relationship existed between GGT and T2D in such complicated clinical settings. To better understand their relationship, the present study developed a liver-specific GGT1-overexpressing mice model to control confounding factors and tested the effects of isolated GGT elevation on GSSG/GSH metabolism, glucose metabolism, and insulin sensitivity.

## 2. Method

### 2.1. Construction of GGT1 Systemic and Liver-Specific Overexpression Vector

For systemic expression, pcDNA3.1-Zeo(+) vector was used. The encoding region of mouse GGT1 was amplified with primers listed below by RT-PCR. For more effective expression of GGT1, two different Kozak sequences were selected and added in different primers (GGT-F1-KOZg: 5′-ACGGGATCCAAGCGCCATGAAGAATCG -GT-3′; GGT-F1-KOZa: 5′-ACGGGATCCAAGCACCATGAAGAATCGGT-3′). Then, the GGT1 cDNA was cloned into the BamHI and XhoI sites of pcDNA3.1-Zeo(+) to generate two different recombinant vectors (pcDNA3.1-ggt1-KOZg and pcDNA3.1-ggt1-KOZa).

pLIVE™ vector, which is designed for liver-specific expression and utilizes a chimeric promoter composed of the mouse minimal albumin promoter and the mouse alpha fetoprotein enhancer II(Mirus Bio Corporation), was selected to construct the liver-specific GGT1 overexpression vector. The pcDNA3.1-ggt1-KOZa was excised with BamHI and XhoI endonucleases and purified by using standard techniques; then, the GGT1 cDNA with Kozak sequence (ACCATGA) was cloned into the BamHI and XhoI sites of pLIVE vector to generate pLIVE-ggt1-KOZa vector. The vector DNAs were prepared by an AxyPrep™ Endo-Free plasma Maxiprep kit.

### 2.2. In Vitro Expression and Enzyme Activity Assays

COS7 cells were cultured in high-glucose DMEM supplemented with 100 U/mL of penicillin, 100 *μ*g/mL of streptomycin, and 10% FBS in 5% CO2, in a 37°C incubator. Approximately 1 × 10^4^ cells were plated and transiently transfected with 0.5 *μ*g of pcDNA3.1-ggt1-KOZg or pcDNA3.1-ggt1-KOZa using Lipofectamine® LTX Reagent (Invitrogen Corp., Carlsbad, CA, USA) according to the manufacturer's protocol. After 48 hours, the cell culture mediums were collected. Then, the cells were scraped off the plate and lysed immediately with PBS followed by determination of protein concentration with a Thermo Scientific Pierce BCA Protein Assay Kit. The culture mediums and the cell lysates were subjected to detect GGT activity using a *γ*-Glutamyl transferase Assay kit (Nanjing Jiancheng Bioengineering Institute, Nanjing, China).

### 2.3. Generation of a Liver-Specific GGT1-Overexpressing Mouse Model

Male C57BL/6 mice were purchased from Chongqing Tengxin Biotechnology Co. Ltd (Chongqing, China). All animals were maintained in a constant 12 h light/12 h dark cycle and fed with a standard rodent chow and water ad libitum. The constructs of pLIVE as a control and pLIVE-ggt1-KOZa vector DNA (100*μ*g/mice) were delivered to the mouse liver using the hydrodynamic tail vein injection procedure according to the instruction provided by manufacturer. To confirm whether the changes in insulin sensitivity in GGT1-L-OE mice were induced by liver-specific overexpression of GGT, GGT1-L-OE mice were administered with 2.5 mg/kg/d GGsTop™ (GGT1-L-OE-sTop mice). For serum GGT activity measurement, mice serum samples were collected at 1, 2, and 3 weeks after injection. For tissue GGT activity measurement, mice were sacrificed at 14 days and the liver, kidney, pancreas, epididymal adipose, and skeletal muscle were quickly frozen in liquid nitrogen and stored at −80°C. Serum and tissue total glutathione (T-GSSG), GSSG, and GSH and total oxidization capacity were assayed at 14 days after vector DNA injection.

To investigate the effects of GGT overexpression on the glucose tolerance and insulin sensitivity, intraperitoneal glucose tolerance test (IPGTT) and insulin tolerance test (ITT) were performed at 14 days after vector DNA injection. For measurement of AKT phosphorylation, 14 days after vector DNA injection, mice were fasted for 6 h and were injected intraperitoneally with or without 0.75 U insulin/kg body weight. Immediately after insulin stimulation, the mouse liver was quickly frozen in liquid nitrogen and stored at −80°C.

### 2.4. IPGTT and ITT

In brief, IPGTT was performed by intraperitoneal injection with 1 g glucose/kg body weight after a 6 h fast, and the blood glucose was measured before and at 15, 30, 60, and 120 minutes after glucose injection. For ITT, mice were injected intraperitoneally with 0.75 U insulin/kg body weight after a 6 h fast and the blood glucose was measured before and at 15, 30, 60, and 120 minutes after insulin injection. Blood glucose concentrations were measured by the Roche Accu-Chek active glucose meter (Roche Diagnostics GmbH, Mannheim, Germany).

### 2.5. Measurement of GGT and Oxidative Stress Markers

Serum and tissue GGT activities were tested by the *γ*-Glutamyl transferase Assay kit (Nanjing Jiancheng Bioengineering Institute, Nanjing, China). The concentrations of T-GSSG, GSSG, and GSH in serum and tissue were measured with the GSH/GSSG assay kit (Beyotime, Beijing, China). As for measurement in tissue lysates, the concentrations of T-GSSG, GSSG, and GSH were normalized by the protein concentrations of tissue lysates. The serum and tissue total oxidization capacities were assayed with a Total Antioxidant Capacity Assay Kit (Beyotime, Beijing, China).

### 2.6. Western Blotting

Tissue lysates were prepared in lysis buffer (Beyotime Institute of Biotechnology, Shanghai, China) on ice in 1.5 mL microtubes for 15 min and centrifuged for 5 min at 12,000*g* at 4°C. The supernatant was collected and protein concentrations were measured using the Thermo Scientific Pierce BCA Protein Assay Kit (Pierce Biotechnology, Rockford, USA); then, the protein samples were stored at −80°C until further examination.

For the Western blot, tissue lysates were subjected to SDS-PAGE and immunoblotting was performed using specific antibodies against AKT (Cell Signaling Technology Inc., Boston, USA), phospho-AKT (Ser473) (Cell Signaling Technology Inc., Boston, USA), and GAPDH (ZSGB-Bio Inc., Beijing, China).

### 2.7. Statistics

Data were expressed as mean ± SEM. Data were analyzed by 1-way ANOVA. *P* values of less than 0.05 were considered statistically significant.

## 3. Results

### 3.1. Evaluation of the Translation Efficiency of GGT1 and Generation of Liver-Specific GGT1-L-OE Mice

**Figure 1:**
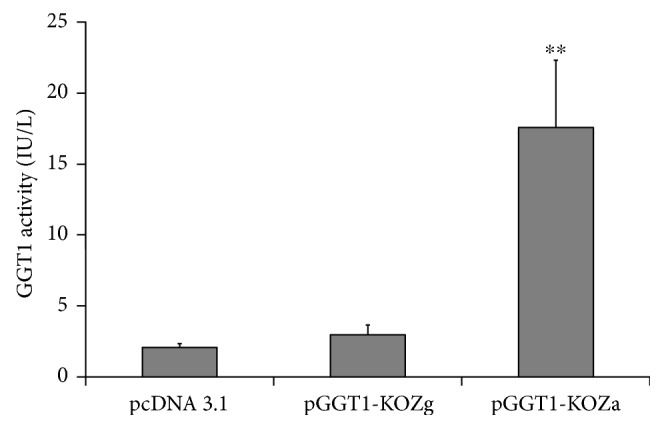
Expression of GGT1 in COS7 cell lines. ^∗∗^*P* < 0.001.

As shown in Figure 1, the GGT activities were significantly increased in the culture mediums and the cell lysates from COS7 cells which were transiently transfected with pcDNA3.1-ggt1-KOZa but not with pcDNA3.1-ggt1-KOZg. These data indicated that the Kozak sequence ACCATGA, which was added in the recombinant vector pcDNA3.1-ggt1-KOZa, promoted more effective translation than the consensus GCCATGA in the vector pcDNA3.1-ggt1-KOZg. Then, the Kozak sequence, ACCATGA, was selected to generate liver-specific GGT1 overexpression vector, pLIVE-ggt1-KOZa.

**Figure 2:**
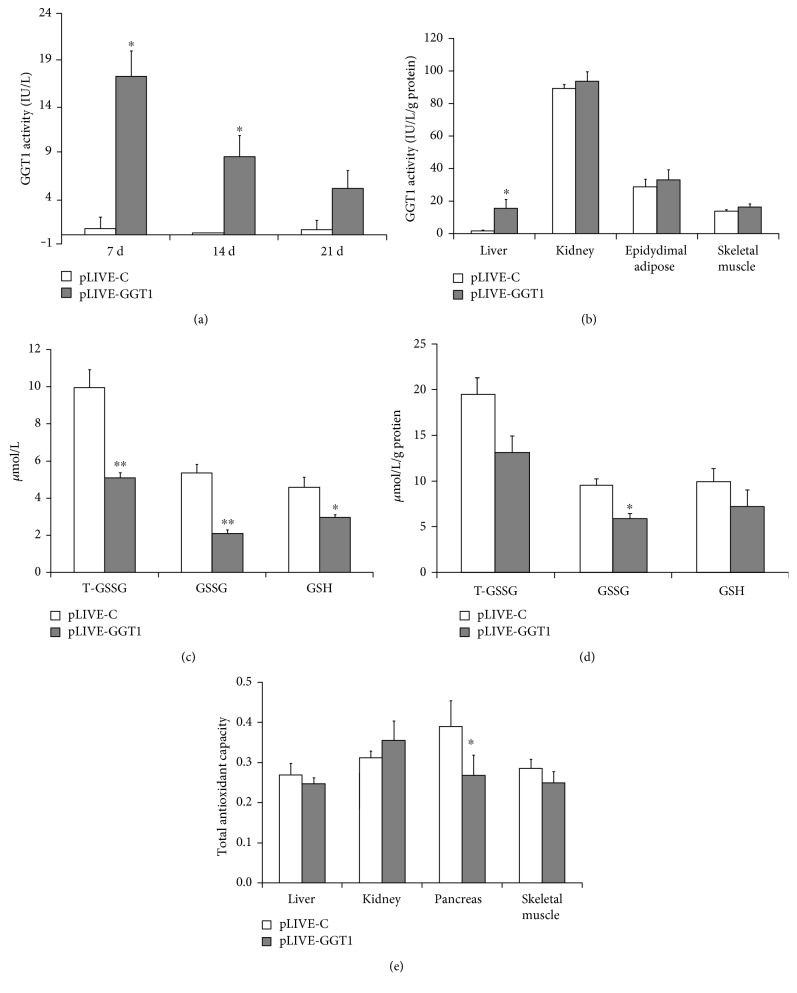
Generation of liver-specific GGT1-overexpressing mice. (a) Serum GGT activities on 7, 14, and 21 days after plasmid delivery through tail vein injection. (b) GGT activities in the liver, kidney, skeletal muscle, and pancreas on the 14 days after plasmid delivery. (c) The concentrations of T-GSSG, GSSG, and GSH in serum. (d) The concentrations of T-GSSG, GSSG, and GSH in liver homogenates. (e) The levels of total antioxidant capacity in the liver, kidney, pancreas, and skeletal muscle. ^∗^*P* < 0.05, ^∗∗^*P* < 0.001.

As shown in Figure 2(a), the serum GGT activities were significantly increased after 7 days of pLIVE-GGT1 vector delivery. The serum GGT activities were decreased by 50% after 14 days of plasmid delivery and by 70% after 21 days of plasmid delivery. So, we designed to explore the effect of liver-specific GGT1 overexpression for a short period of 14 days on the glucose tolerance and insulin sensitivity. 

The liver, pancreas, kidney, epididymal adipose, and skeletal muscle of GGT1-L-OE mice were lysed and subjected to test GGT1 activities on the 14 days after plasmid delivery. As expected, the GGT enzymatic activities were significantly increased in the liver but not in the kidney, pancreas, epididymal adipose, or skeletal muscle (Figure 2(b)).

The levels of T-GSSG, GSSG, and GSH were reduced in serum and liver homogenates. The total GSSG levels were significantly reduced by 50% in serum and by 30% in liver homogenates from GGT1-L-OE mice compared with those from control mice. Compared with those in control mice, serum GSH levels were decreased by 30% in GGT1-L-OE mice. But there was no significant decrease in liver homogenates from GGT1-L-OE mice compared with those from control mice. Similarly, no significant changes in the total antioxidant capacity were observed in the liver from GGT1-L-OE mice compared with that from control mice.

### 3.2. Liver-Specific Overexpression of GGT Had No Effect on the Glucose Tolerance

**Figure 3:**
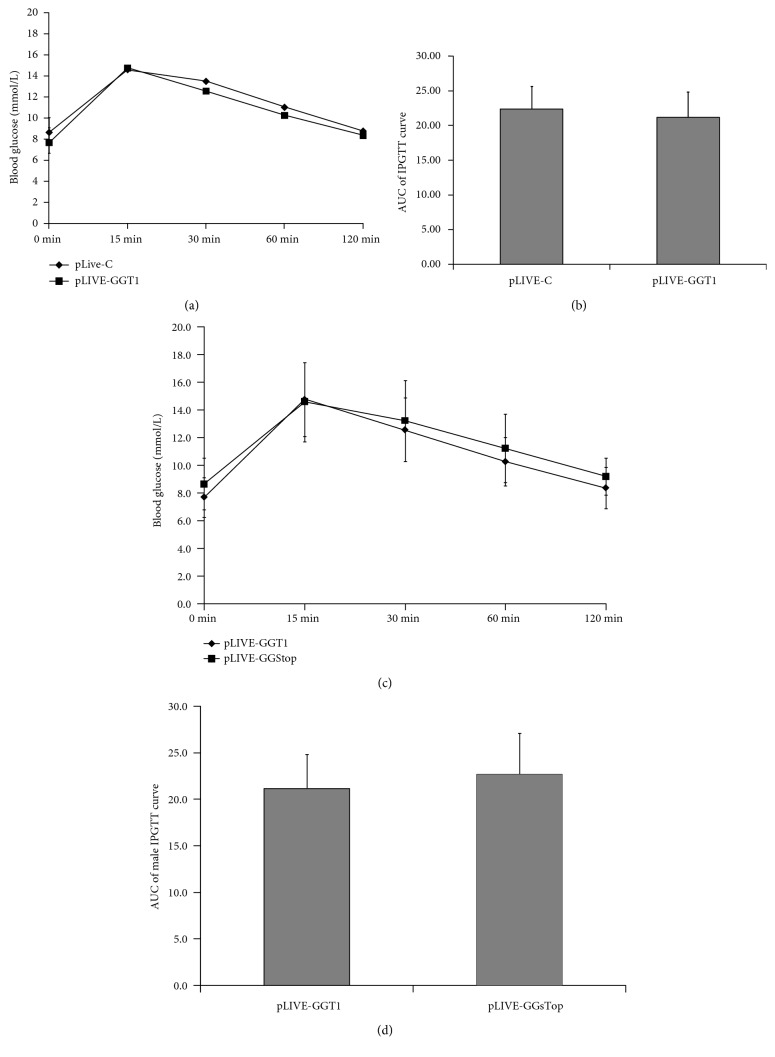
Overexpression of GGT1 in the liver had no effect on glucose tolerance. (a) IPGTT was performed and the blood glucose was measured at the indicated time points (*n* = 13–17); (b) AUC of the IPGTT curve. (c) IPGTT was performed in GGT1-L-OE mice and GGT1-L-OE-sTop mice which are treated with GGsTop, a highly selective GGT inhibitor, and the AUC was evaluated (d).

As shown in Figures 3(a) and 3(b), the levels of blood glucose were slightly increased before and at 30 and 60 minutes after glucose challenge in GGT1-L-OE mice than those in the control mice, but the differences of the levels of blood glucose and the AUC of IPGTT curve between the two groups were not statistically significant. Furthermore, the levels of blood glucose were slightly decreased before and at 30, 60, and 120 minutes after glucose challenge in GGT1-L-sTop mice which were treated with GGsTop, than those in the control mice, but there were no statistical significances between the two groups. These results indicated that liver-specific overexpression of GGT in two weeks had no effect on the glucose tolerance in mice without metabolic diseases.

### 3.3. Liver-Specific Overexpression of GGT Increased Insulin Sensitivity in C57BL/6 Mice

**Figure 4:**
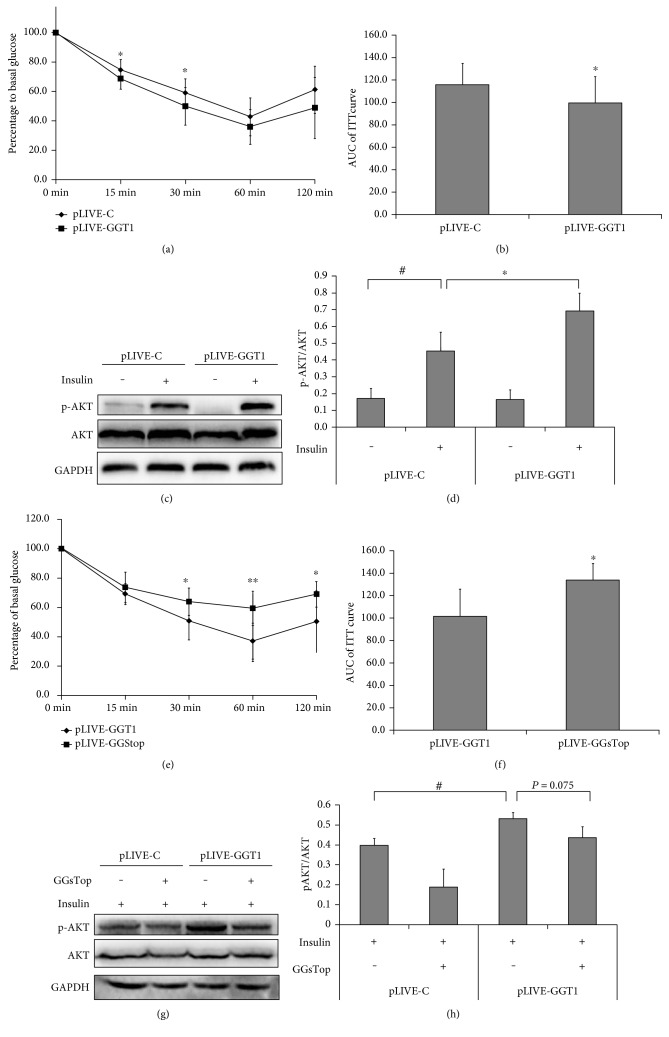
Overexpression of GGT1 in the liver increased insulin sensitivity. (a) ITT was performed and the blood glucose was measured at the indicated time points (*n* = 13–17); (b) AUC of blood glucose after ITT. (c) The liver from fasted C57BL/6 mice injected with or without 0.75 U insulin/kg body weight for 15 min was subjected to Western blotting using a rabbit anti-phosphor-AKT (Ser473) polyclonal antibody or rabbit anti-total AKT polyclonal antibody. (d) Fold changes of phosphor-AKT versus total AKT relative to the basal levels in GGT1-L-OE and control mice were quantified by densitometry. (e) ITT was performed in GGT1-L-OE mice and GGT1-L-OE-sTop mice which were treated with GGsTop, a highly selective GGT inhibitor, and the AUC was counted (f). (g) Liver homogenates were immunoblotted with anti-phosphor-AKT (Ser473) antibody or anti-AKT antibody. (h) The relative expression of phosphor-AKT was presented by setting the mean density of blots. ^∗^*P* < 0.05, ^∗∗^*P* < 0.001, ^#^< 0.05.

Furthermore, we investigated whether specific overexpression of GGT in the liver affected whole-body insulin sensitivity by ITT. As shown in Figures 4(a) and 4(b), blood glucose concentrations were decreased more in GGT1-L-OE mice during ITT when compared with those in the control mice. The levels of blood glucose were decreased significantly at 15 and 30 minutes after insulin administration. Moreover, the AUC of ITT curve in GGT1-L-OE mice was significantly decreased by 15% when compared with that in the controls (Figure 4(b)). Similarly, the levels of phosphorylated AKT at Ser473, induced by insulin, were significantly increased in the liver lysates of GGT1-L-OE mice compared with those in the liver lysates of the controls (Figures 4(c) and 4(d)).

To confirm whether the increased insulin sensitivity in GGT1-L-OE mice was induced by specific overexpression of GGT in the liver, GGsTop was used. As expected, blood glucose concentrations decreased less in the GGT1-L-OE-sTop mice during ITT compared with those in the GGT1-L-OE mice. The differences of blood glucose levels were significant at 30, 60, and 120 minutes after insulin administration. Also, the AUC of ITT curve in GGT1-L-OE-sTop mice was significantly increased by approximately 30% when compared with that in the GGT1-L-OE mice (Figure 4(b)). The levels of phosphorylated AKT at Ser473, induced by insulin, were decreased by about 20% in the liver lysates of the GGT1-L-OE-sTop mice compared with those in the liver lysates of the GGT1-L-OE mice, although no statistical significance was reached (*P* = 0.075). These results suggested that specific overexpression of GGT in the liver might increase insulin sensitivity in C57BL/6 mice.

## 4. Discussion

GGT transgenic mice models had been used to study osteoporosis and tumor metastasis [14, 15], but regrettably, no study focused on glucose metabolism. In the present study, we successfully developed a short-term liver-specific GGT1-overexpressing mice model and found that increased activity of GGT in the liver and circulation enhanced the catabolism of GSSG/GSH. GGT elevation, in two weeks, had no effects on the glucose metabolism but surprisingly might increase insulin sensitivity in the liver of the transgenic mice. Such results were quite different from those observed in epidemiologic studies that elevated GGT activity increased the risk of T2D.

GGT elevation was common in T2D and other clinical conditions such as obesity, NAFLD, prediabetes, and metabolic syndrome [5]. In such conditions, GGT elevation usually was accompanied by increased levels of ferritin [9, 12], uric acid [16], and markers of chronic inflammation such as CRP [12] and IL-6 [13]. Besides GGT, all of the above factors have been reported to be associated with increased risk of T2D [17–19]. Iron overload initiated pro-oxidant reaction and was involved in multiple pathological mechanisms [17, 20]. GGT might exert effects in cooperation with iron. For example, our previous study observed that GGT might affect T2D risks by synergetic action with increased serum ferritin rather than GGT alone [9]. Interestingly, another study of gestational diabetes (GDM) among a large cohort of mothers in California also showed that GGT only predicted GDM in women with top tertile of HOMA-IR before pregnancy, suggesting interaction between GGT and HOMA-IR [21]. The latter was a well-established correlate of serum ferritin. In this study, we purely increased GGT activity and neglected any potential interactive effects with other aforementioned factors. This could be an explanation for the conflicting conclusions between previous epidemiologic studies and this experimental study.

This study observed that GGT overexpression reduced the levels of GSSG/GSH but had little effects on total antioxidant capacity, suggesting its influence on ROS being mild. To our surprise, this study showed that increased GGT activity enhanced insulin-induced AKT phosphorylation at Ser473 and insulin-induced glucose uptake. GGT is critical to maintain cysteine homeostasis. It has been reported that L-cysteine increased phosphatidylinositol 3,4,5-trisphosphate (PIP3), a positive regulator of phosphorylation of AKT, in 3T3-L1 adipocytes, and subsequently enhances glucose utilization by the activation of phosphoinositide 3-kinase and phosphorylation of AKT [22, 23]. So, tt is plausible to deduce that liver-specific GGT overexpression increased AKT phosphorylation through cysteine-mediated mechanisms. Published studies already suggested that GGT elevation could just be one of the corresponding changes to other offending factors such as oxidative stress. GGT elevation in T2D could be a compensation mechanism but not strong enough to ameliorate increased oxidative stress [20, 24]. GGT might actually play a protective role against the harmful impact by the aforementioned factors. When GGT elevation is sufficient, like in the GGT1-L-OE mice from this study, it might exert beneficial effects on insulin sensitivity via AKT-mediated mechanisms.

There were some limitations in the present study. The major one was the short-term duration of GGT overexpression. Hydrodynamic delivery has been proved to be an effective method for targeted gene transfection in animals. It works by a rapid injection of DNA solutions via the tail vein, leading to significant transgene expression in multiple organs, especially in the liver. The expression of some transfected genes by this method could last for several months [25]. While in this study, GGT elevation could only persisted for less than 3 weeks. We had tried different buffer solutions and plasmid concentrations but failed to extend the expression time. A future study should employ other transgenic methods to evaluate the effects of prolonged GGT overactivity on insulin sensitivity. Secondly, the GGT-mediated insulin sensitivity improvement was observed only in normal C57BL/6 mice but not in mice with metabolic diseases such as obesity or T2D. Thus, it is not known whether elevated GGT activity influences glucose metabolism and/or insulin sensitivity in T2D or metabolic syndrome mice models. Further studies are needed to confirm such relationship in those mice models.

In conclusion, the presented study demonstrated that GGT elevation in a short term had no effects on glucose tolerance but could promote insulin sensitivity in male C57BL/6 mice. These results supported the notion that GGT elevation could be a protective factor rather than an offending factor to T2D.
